# Azelaic Acid Exerts Antileukemia Effects against Acute Myeloid Leukemia by Regulating the Prdxs/ROS Signaling Pathway

**DOI:** 10.1155/2020/1295984

**Published:** 2020-12-23

**Authors:** Dongdong Zhang, Ziyi Luo, Yanxia Jin, Yanling Chen, Tian Yang, Qian Yang, Balu Wu, Yufeng Shang, Xiaoyan Liu, Yongchang Wei, Fuling Zhou

**Affiliations:** ^1^Department of Hematology, Zhongnan Hospital, Wuhan University, No. 169 Donghu Road, Wuchang District, Wuhan, 4300071 Hubei Province, China; ^2^Department of Oncology, Xiangyang No. 1 People's Hospital, Hubei University of Medicine, Xiangyang, Hubei 441000, China; ^3^Department of Radiation and Medical Oncology, Zhongnan Hospital, Wuhan University, Wuhan 430071, China

## Abstract

Acute myeloid leukemia (AML) is a hematological malignancy with a poor prognosis attributed to elevated reactive oxygen species (ROS) levels. Thus, agents that inhibit ROS generation in AML should be exploited. Azelaic acid (AZA), a small molecular compound, can scavenge ROS and other free radicals, exerting antitumor effects on various tumor cells. Herein, this study evaluated the antileukemic activity of AZA against AML via regulation of the ROS signaling pathway. We found that AZA reduced intracellular ROS levels and increased total antioxidant capacity in AML cell lines and AML patient cells. AZA suppressed the proliferation of AML cell lines and AML patient cells, expending minimal cytotoxicity on healthy cells. Laser confocal microscopy showed that AZA-treated AML cells surged and ruptured gradually on microfluidic chips. Additionally, AZA promoted AML cell apoptosis and arrested the cell cycle at the G1 phase. Further analysis demonstrated that peroxiredoxin (Prdx) 2 and Prdx3 were upregulated in AZA-treated AML cells. *In vivo*, AZA prolonged survival and attenuated AML by decreasing CD33^+^ immunophenotyping in the bone marrow of a patient-derived xenograft AML model. Furthermore, mice in the AZA-treated group had an increased antioxidant capacity and Prdx2/Prdx3 upregulation. The findings indicate that AZA may be a potential agent against AML by regulating the Prdxs/ROS signaling pathway.

## 1. Introduction

Acute myeloid leukemia (AML) is one of the most common hematological malignancies with a rapidly progressive and poor prognosis. Larger numbers of blasts accumulate in the bone marrow and infiltrate other tissues, thus inhibiting hematopoietic functions and inducing subsequent hemorrhage and severe infection. The annual mortality rate is 2.2 per 100,000 [1]. High-dose induction chemotherapy for inhibiting leukemic blast proliferation in the acute stage and consolidation chemotherapy during the remission stage remain the main methods of treating AML; the curative treatment for AML is successful allogeneic stem cell transplantation after achieving complete remission. However, some patients cannot tolerate the toxic adverse effects of chemotherapeutic drugs and experience chemotherapeutic resistance and severe adverse reactions, such as severe infection, that can lead to treatment failure and result in death.

Reactive oxygen species (ROS) are small short-lived oxygen-containing molecules that regulated many processes, such as cell growth and death, inflammation, stem cell renewal, tumorigenesis, oxygen sensing, angiogenesis, and immune responses [2, 3]. Elevated ROS levels are a major cause of DNA damage and mutation, triggering malignant cell transformation and promoting cancer initiation [4], including the initiation and progression of inherited and sporadic human leukemias [5]. ROS are frequently elevated in cancer cells [6]. Likewise, AML patients have significantly increased ROS levels and a lower total antioxidant capacity (T-AOC) [7]. Overproduction of NADPH oxidase-derived ROS and Ras-induced ROS can promote the proliferation of AML blasts [8, 9], thus accelerating AML progression [10]. In addition, increased intracellular ROS levels are concomitant with thioredoxin (Trx) and 8-hydroxydeoxyguanosine overexpression, which is associated with AML relapse [7]. Excessive ROS trigger activation of the oncogene, c-Jun activation domain-binding protein 1 (*Jab1*), in AML patient relapse, and the upregulated Jab1 can regulate Trx by binding to Trx1, which contributes to the poor survival [11]. Additionally, increased ROS levels have been correlated with phenotypic change in hematopoietic stem cells (HSCs) and loss of HSC quiescence, and excessive oxygen limits the lifespan of HSCs by regulating the ROS-p38 MAPK pathway [12].

The roles of ROS depend on their intracellular levels. Low ROS levels are required to initiate and promote tumor growth; moderate ROS levels are involved in the inflammatory response, while high ROS levels contribute to apoptosis and autophagy [13]. Both ROS-elevating and ROS-eliminating strategies have been developed to treat cancer [14]. Over the past several years, ROS-elevating strategies have been predominantly used in clinics, including agents targeting the Prdx2 and Prdx3 to treat AML [15–17]. However, overproduction of ROS induced by chemotherapeutic drugs increases oxidative stress, which can lead to therapeutic resistance and therefore help to drive tumor recurrence [18, 19]. AML patients with FMS-like tyrosine kinase 3 (FLT3) mutations have high relapse rates because FLT3 induces elevated ROS levels [20, 21]. In addition, increased ROS levels accompanied by Trx1 and Jab1 overexpression are correlated with recurrence and poor survival in AML patients [11]. Furthermore, elevated ROS levels greatly contribute to immunosuppression in the tumor microenvironment [19]. Overproduction of ROS triggers the dysfunction of natural killer and T-cells and inhibits the cytotoxicity of effector cells [22, 23]. Therefore, ROS-eliminating strategies have emerged as a promising approach to treating AML.

Azelaic acid (AZA) is a natural, nontoxic, saturated, nine-carbon dicarboxylic acid that was first recognized as a secondary metabolite in fungal infections with *Malassezia* [24]. AZA, as a competitive inhibitor of tyrosinase [25] and other oxidoreductases, has hypopigmentation and anti-infective properties and is commonly used to treat skin disorders such as melasma and acne [26]. Prior studies demonstrated that AZA can scavenge ROS and inhibit the generation and action of oxygen radicals [27, 28]. AZA can also reversibly inhibit cytochrome-P450 reductase and respiratory chain enzymes [29]. Furthermore, AZA exhibits antitumor effects on several tumor cells, such as lentigo maligna [30], malignant melanoma [31], lymphoma [32], and human T lymphotropic virus 1- (HLTV-1-) infected T-cell leukemia [33], by inhibiting Trx reductase activity, ROS generation, and DNA synthesis in tumor cells [28, 31, 33]. A previous study showed that AZA could suppress AML cell proliferation and sensitize AML cells to chemotherapy [34]. However, the exact mechanism of AZA on AML cells remains unknown. Therefore, in the present study, we examined the antileukemia activity of AZA and further explored its molecular basis.

## 2. Materials and Methods

### 2.1. Materials

DMSO (Cat# D2650) and AZA (Cat# 95054) were purchased from Sigma (USA). PrimeScript™ RT reagent kit with gDNA Eraser was from Takara (Cat# RR047A). Annexin V-FITC Apoptosis Detection Kit was from KeyGEN Biotech (Cat# KGA105-KGA108, China). Cell Cycle Staining Kit was from MultiSciences Biotech (Cat# CCS012, China). Antibodies to the following proteins were used: Prdx3 was from CUSABIO (Cat# CSB-PA003861, China); *β*-actin (Cat# 14395-1) and Prdx2 (Cat# 10545-2) were from ProteinTech (USA).

### 2.2. Cell Culture

AML cell lines U937, HL60, THP-1, and Molm-13 cells were grown in RPMI-1640 medium supplemented with 10% fetal bovine serum and penicillin/streptomycin at 37°C. Different types of AML patient primary cells (AML-PC) according to the FAB classification and healthy peripheral mononuclear cells (PBMCs) were isolated by Ficoll-Hypaque gradient centrifugation followed by the manufacturer's recommendation (TBD sciences, Cat# HY2015, China); the detailed AML patient information can be found in Table S1. Importantly, all subjects were given written informed consent in accordance with the recommendations of the Ethics and Scientific Committee of Zhongnan Hospital of Wuhan University and the Declaration of Helsinki; the Ethics Committee of Wuhan University approval number is 2018278.

### 2.3. Measurement of ROS

Intracellular ROS levels were measured using the Reactive Oxygen Species Assay Kit (Beyotime, Cat# S0033, China). Briefly, HL60, U937, THP-1, and AML-PC cells were treated with AZA for 24 h, and the collected cells were then washed with serum-free RPMI-1640 and incubated with 2,7-dichlorodihydrofluorescein diacetate (DCF-DA) at the concentration of 5 *μ*M for 20 minutes at 37°C to assess ROS-mediated oxidation of the fluorescence compound DCF-DA. ROS levels were determined by detecting the fluorescence intensity of the oxidized DCF cytometer, and the flow cytometry analysis results were quantified by using CytExpert2.0 software.

### 2.4. ROS-Related Index Analysis

HL60, U937, and Molm-13 cells were pretreated with 5 mM AZA for 24 h; cells were collected and washed twice by PBS. Cells were lysed by using the ultrasonic wave breaking, and cell homogenate was centrifuged for 15 minutes at 12,000 rpm at 4°C. Thereafter, supernatant was harvested and stored at -20°C. In vivo experiment, peripheral blood of mice was centrifuged for 15 minutes at 12,000 rpm at 4°C in an anticoagulant tube and the serum was collected from liquid supernatant. The harvested cell supernatant and serum were prepared for the following study. The total antioxidant capacity (T-AOC, Cat# A105), superoxide dismutase (SOD, Cat# A001) activity, glutathione peroxidase (GSH-Px, Cat# A006) content, and malondialdehyde (MDA, Cat# A003) levels were assayed according to the manufacturer's protocol as described previously (Nanjing Jiancheng Bioengineering Institute, China) [11].

### 2.5. Analysis of Cell Viability

Cell viability was measured by using a Cell Counting Kit-8 (Dojindo, Cat# JE603, Japan). HL60, THP-1, Molm-13, and different types of AML-PC cells were seeded in a 96-well plate and treated with different concentrations of AZA for 24 h. Thereafter, 10 *μ*L CCK8 was added to each plate and incubated for additional 2 h. The cell viability was measured by reading the absorbance at 450 nm.

### 2.6. Cytotoxicity Assays at the Single-Cell Level

To observe the AML cell viability after AZA treatment, THP-1 cells transfected with GFP were used in our study and a DAPI stain was used as a control for indicating all cell numbers in the field. When the GFP^+^-THP-1 cell was dead, the green fluorescence disappeared. The cell viability can be assayed by comparing the green fluorescence intensity between two groups.

To detect the cytotoxic effect of AZA on AML cells, a microfluidic chip was designed to capture the cells and allowing the injection of AZA. The clip size was designed according to the cell size as described previously [35, 36]. Briefly, HL60 cells were injected into the chip and fixed in one inlet, and 5 mM AZA was slowly injected from another inlet. The dynamic changes in the cell morphology after AZA treatment at different time points and fields were observed under microscopy (Nikon).

### 2.7. Measurement of Mitochondrial Membrane (MMP)

Early apoptosis analysis was measured by using the Mitochondrial Membrane Potential Assay Kit with JC-1 (Beyotime, Cat# C2006, China). JC-1 aggregates in the matrix of mitochondria to form a polymer (J-aggregates) and can emit red fluorescence at normal MMP but gets converted to monomer when MMP decreased and then can emit green fluorescence. HL60 cells were treated with 5 mM AZA for 24 h; cells were collected and stained with JC-1 dye. The change in mitochondrial membrane potential was analyzed by flow cytometry.

### 2.8. Cell Apoptosis and Cell Cycle Assays

The apoptotic rate of U937 cells was detected with the Annexin V/FITC Apoptosis Detection Kit. U937 cells were seeded in 6-well plates and treated with 5 mM AZA for 24 h. The collected cells were washed twice by PBS and stained with 5 *μ*L Annexin V-FITC and 5 *μ*L propidium iodide and then incubated for 5 minutes at 25°C. The apoptotic rate was measured by calculating the percentage of FITC and PI-positive cells with flow cytometry.

Cell cycle was measured by using a Cell Cycle Staining Kit. U937 cells were pretreated and collected as above described. Cells were then fixed by 1 mL staining buffer and incubated with 10 *μ*L propidium iodide for 30 minutes. The number of cells in different phases of the cell cycle was analyzed by flow cytometry. Data were analyzed by using CytExpert2.0 software.

### 2.9. Protein Mass Spectrometry Analysis (LC-MS)

THP-1 cells were treated with 5 mM AZA for 24 h, and total proteins were extracted by RIPA buffer. Then, the peptides from the AZA-treated group and the control group were labeled with isotopomeric dimethyl label. The labeled samples were analyzed by using a hybrid Quadrupole-TOF Mass Spectrometer as described previously [37] (TripleTOF 5600, AB Sciex Instruments). The raw LC-MS data was accessible on PeptideAtlas, and the direct URL is http://www.peptideatlas.org/PASS/PASS01499.

### 2.10. RNA Isolation, cDNA Preparation, and Quantitative PCR

THP-1 and Molm-13 cells were treated with 5 mM AZA for 24 h. The total RNA was isolated, and the reverse transcription for cDNA was performed by using the PrimeScript™ RT reagent kit with gDNA Eraser. SYBR-GREEN qPCR was performed to measure the peroxiredoxin 2 (Prdx2) and peroxiredoxin 3 (Prdx3) expression using the SYBR GREEN MIXTURE kit according to the manufacturer's recommendations. The expression level was analyzed using the 2^-*ΔΔ*CT^ approach. The primers are listed as follows: GAPDH, sense, 5′-TGATGACATCAAGAAGGTGGTGAA-3′, antisense, 5′-TCCTTGGAGGCCATG TGGGCCAT-3′; Prdx3, sense, 5′-GCCGCTCTGTGGATGAGACT-3′, antisense, 5′-CCAGCTGGGCACACTTCC-3′; Prdx2, sense, 5-GTGTCCTTCGCCAGATCACT-3′, antisense, 5-ACAAACTTCCCCATGCTCGT-3′.

### 2.11. Western Blotting Analysis

Molm-13, THP-1, and AML-PC cells were seeded in 6-well plates and treated with 5 mM AZA for 24 h. Total proteins were extracted by RIPA and 10 mM PMSF. Thereafter, protein concentration was determined by the BCA protein assay kit (ThermoFisher, USA). The proteins (20 *μ*g) were resolved by SDS gel electrophoresis and transferred to PVDF membranes. The membranes were blocked by 5% nonfat milk followed by probing with primary and secondary antibodies.

### 2.12. Animal Experiment

The B-NSG mice (female, 16-18 g, 5 weeks) were obtained from the Animal Research Center of Wuhan University. AML-PC cells were used to construct the model which were isolated from a hyperleukocytic AML patient sample after undergoing leukapheresis by using a Fresenius COM.TEC machine. Mice were given 1.5 Gy X-ray and injected with 1.0 × 10^7^ human AML cells/per mouse intravenously within 24 h for the development of leukemic disease. The patient-derived xenograft (PDX) AML model was successfully established when AML-PC cells could be observed on the peripheral blood (PB) and bone marrow (BM) smears. Then, mice were randomly divided into two groups with 6 mice in each group. The mice in the AZA group were treated with 10 mg/kg AZA by intraperitoneal injection (200 *μ*L, every three days, *n* = 8), while the control group (*n* = 8) received saline with the same volume and frequency. At the end of experiments, mice were sacrificed and the tissues were harvested for further study. Importantly, all animal studies were approved by the Institutional Animal Care and Use Committee of Wuhan University (2017048).

### 2.13. Smear Analysis and Immunohistochemistry

After injecting mice with AML-PC cells for one week, we randomly selected one mouse which was humanly killed, and its PB and BM were harvested. PB and BM smears were stained with Wright's stain and observed microscopically to measure the proportion of leukemia cells and determine whether the PDX model was constructed successfully [38].

Tissues collected from the mice were fixed, trimmed, processed, dewaxed, and rehydrated, then under pretreated for antigen retrieval in citrate buffer at pH 6.0 at 100°C for 30 minutes. Thereafter, tissues were blocked with primary antibodies (Prdx2 and Prdx3 antibodies, 1 : 100) overnight, then probed with secondary antibodies. Images were photographed using a Nikon microscope at the Hematology Department, Wuhan University, Zhongnan Hospital, Wuhan, China.

### 2.14. Bioinformatics and Statistical Analysis

Differentially expressed proteins were identified via LC-MS, annotated by WEGO analysis (http://wego.genomics.org.cn/), and analyzed with R code by creating a heat map. The protein-protein interaction networks were analyzed using STRING v11.0 (https://string-db.org/cgi/network). Data are presented as the means ± standard deviations and were analyzed via Student's *t*-test and one-way analysis of variance using GraphPad Prism 7 and IBM SPSS. *P* < 0.05 was considered statistically significant.

## 3. Results

### 3.1. AZA Decreased Intracellular ROS Levels and Increased Antioxidant Capacity

**Figure 1:**
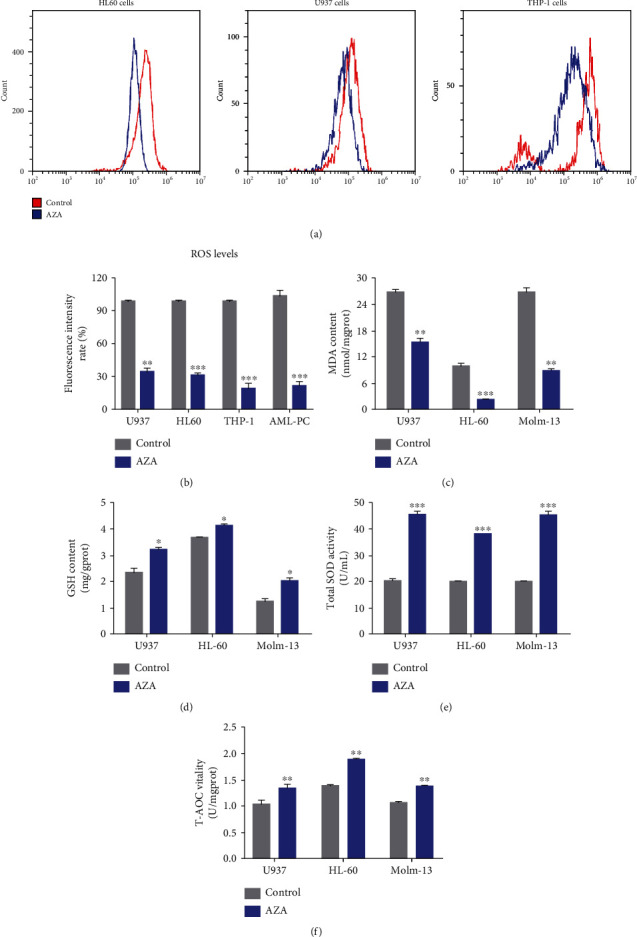
AZA decreased intracellular ROS levels and increased the antioxidant capacity. (a) U937, HL60, and THP-1 cells were treated with 5.0 mM c–AZA for 24 h. The collected cells were stained with DCF-DA, and ROS levels were analyzed by flow cytometry. (b) Statistical analysis of the intracellular ROS levels after AML cell lines and AML patient cells were treated with 5 mM AZA for 24 h. (c–f) U937, HL60, and Molm-13 cells were treated with 5.0 mM AZA for 24 h. The cell homogenates were harvested, and ROS-related indices were tested with kits as described in the methods. (c) MDA; (d) GSH; (e) SOD; (f) T-AOC. Each experiment was repeated three times, ^∗^*P* < 0.05, ^∗∗^*P* < 0.01, and ^∗∗∗^*P* < 0.001.

Prior studies demonstrated that AML patients had elevated intracellular ROS levels and AZA could scavenge the ROS [28]. We detected the ROS levels and ROS-related indices in HL60, THP-1, and U937 cells and human AML cells after treatment with AZA. As expected, AZA markedly decreased the intracellular ROS levels in the AML cell lines and AML patient cells (Figures 1(a) and 1(b)). Furthermore, in the AZA-treated cell homogenate, the oxidative injury indexes, such as the MDA content (Figure 1(c)), were decreased, while the antioxidant injury indexes, such as the SOD and GSH activity and the total antioxidant capacity, were significantly increased (Figures 1(d)–1(f)).

### 3.2. AZA Exhibited Cytotoxicity against AML Cells

**Figure 2:**
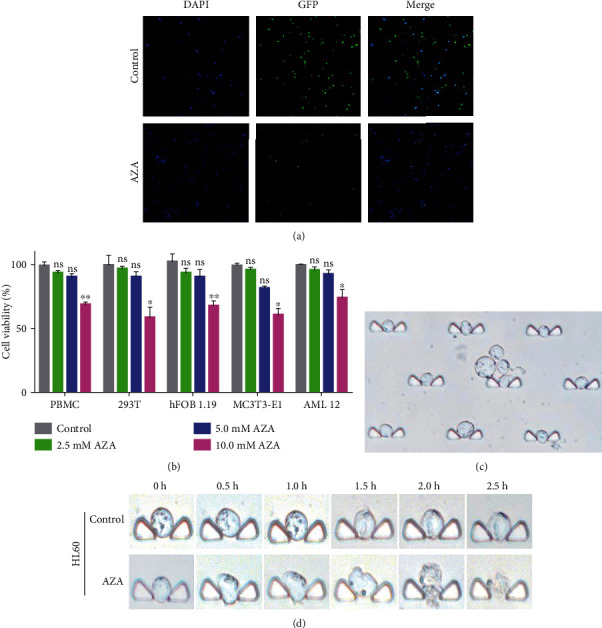
AZA inhibited AML cell proliferation and had a little toxic effect on healthy cells. (a) Molm-13 GFP^+^ cells were treated with 5 mM AZA for 24 h and then observed under a fluorescence microscope; DAPI was used as a control. Magnification: ×40. (b) Healthy cell lines such as PBMC, 293T, hBOT 1.19, MC3T3-E1, and alpha mouse live cells were treated with different concentrations of AZA for 24 h. Cell viability was measured by the CCK-8 method. (c) HL60 cells were trapped on a microfluidic chip and observed under a microscope. (d) HL60 cells were treated with 5 mM AZA on the microfluidic chip, and the cell motion morphology was observed at the single-cell level under a microscope. Magnification: ×60. Each experiment was repeated three times, ^∗^*P* < 0.05, ^∗∗^*P* < 0.01, and ^∗∗∗^*P* < 0.001.

In our previous study, we have identified that AZA had an antiproliferative effect on different AML cell lines, the IC50 value of AZA for 24 h was ranged from 3.4 to 7.2 mM, and the median *IC50* value was approximately 5 mM [34], so we chose the concentration of 2.5, 5.0, and 10.0 mM for our present cytotoxicity assay. AZA markedly suppressed AML cell lines and different types of AML patient cell proliferation dose-independently as described in our previous study (Fig. S1). Additionally, GFP^+^ Molm-13 cells revealed hypofluorescence after treatment with 5 mM AZA for 24 h compared to the control group; this result also indicated AZA could inhibit AML cell viability (Figure 2(a)). However, the same concentration of AZA had little toxicity on PBMC and other healthy cell lines such as 293T, hFOB 1.19, MC3T3-E1, and AML 12 cells (Figure 2(b)). Even so, we believe that more experiments would be required to show that AZA is really more toxic to AML cells compared to hematopoietic stem and progenitor cells.

To observe the cytotoxicity of AZA on AML cells at the single-cell level, we designed a microfluidic chip that can trap cells and injected AZA into the chip from two opposite directions (Figure 2(c)). The HL60 cell morphology became swollen, apoptotic bodies were detected, and the cells ultimately showed lysis after continuous AZA treatment on the microfluidic chips (Figure 2(d)).

### 3.3. AZA Promoted AML Cell Apoptosis

**Figure 3:**
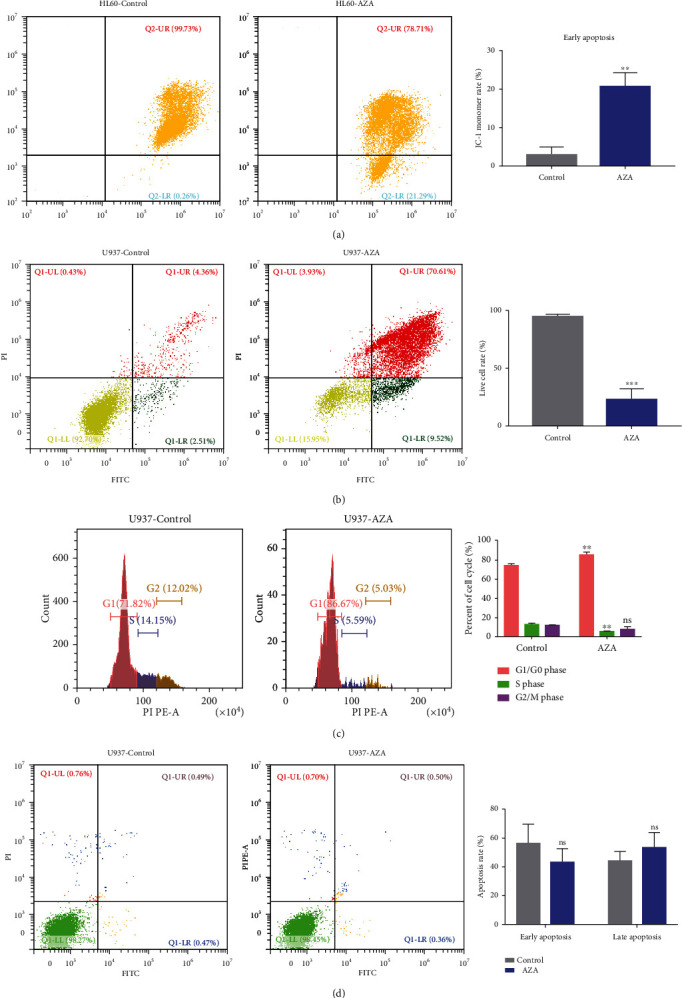
AZA promoted AML cell apoptosis. (a) HL60 cells were treated with 5.0 mM AZA for 24 h. The collected cells were stained with JC-1 dye, and the MMP was analyzed by flow cytometry. (b) U937 cells were treated with 5.0 mM AZA for 24 h. The collected cells were stained with Annexin V and PI. Cell apoptosis rate was analyzed by flow cytometry. (c) Cell cycle was analyzed by flow cytometry after U937 cells were treated with 5.0 mM AZA for 24 h. (d) Cell apoptosis rate was analyzed by flow cytometry after healthy PBMCs were treated with 5.0 mM AZA for 24 h. Each experiment was repeated three times, ^∗∗^*P* < 0.01 and ^∗∗∗^*P* < 0.001. ns: no significance.

Loss of MMP is common in the early stages of apoptosis. AZA induced a significant loss of MMP as measured by the percentage of JC-1 monomer cells (Figure 3(a)). AZA markedly increased the percentage of Annexin-V/FITC-positive cells (Figure 3(b)). Furthermore, the cell cycle was arrested at the G1/G0 phase after AZA treatment (Figure 3(c)). However, the same AZA concentration had no toxicity on PBMC isolated from healthy people (Figures 3(d) and S2).

### 3.4. AZA Upregulated Prdx2 and Prdx3 in AML Cells

**Figure 4:**
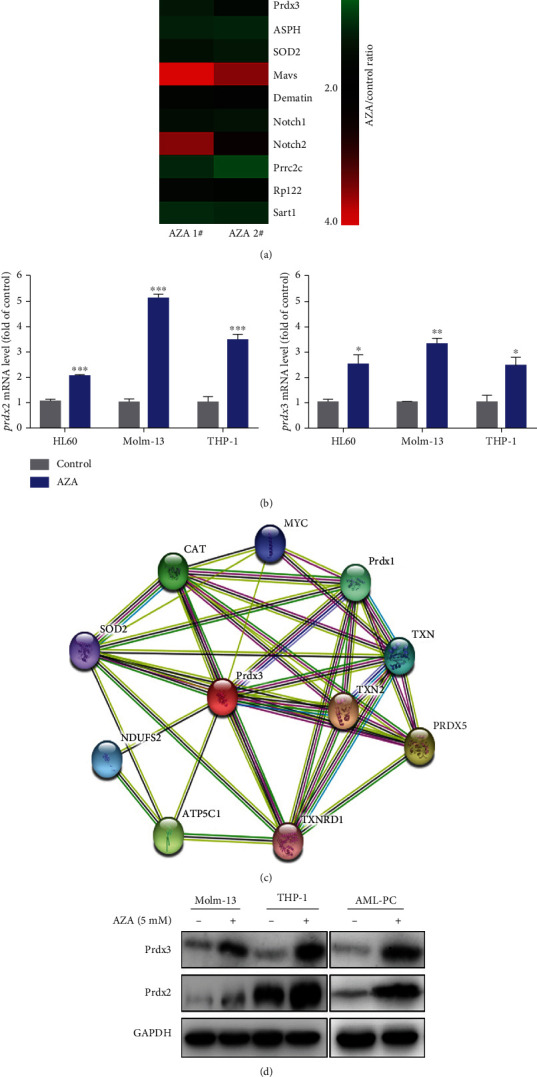
AZA upregulated Prdx2 and Prdx3 expression. (a) Differentially expressed proteins (DEPs) were identified by LC-MS/MS after AZA treatment. Then, DEPs were annotated by WEGO analysis; the changes of DEPs involved in antioxidant activity and immune response were shown on a heat map. (b) The RNA expression levels of Prdx2 and Prdx3 in Molm-13 and THP-1 cells after AZA treatment and their detection by qPCR. (c) The interaction between Prdx3 and other proteins was analyzed by STRING. (d) The protein expression levels of Prdx2 and Prdx3 in Molm-13, THP-1, and AML patient primary cells (AML-PC) after AZA treatment and their detection by western blot.

To understand the molecular basis for the antileukemic activity of AZA, we performed LC-MS analysis. Differentially expressed proteins (DEPs) were identified after AZA treatment. The DEPs were then annotated by WEGO analysis as described previously [38]. Five hundred and twenty-eight DEPs were annotated into three areas: cellular components, molecular functions, and biological processes. The potential DEPs involved in antioxidant activity and immune response were analyzed by a heat map (Figure 4(a)). LC-MS data showed that AZA upregulated Prdx2 and Prdx3 with approximately 2.0-fold higher Prdx3 expression in AZA-treated THP-1 cells than in the control group. The Prdx system played a crucial role in decreasing intracellular ROS levels and maintaining the redox balance [39]. Prdx2 and Prdx3 were the main ROS-scavenging antioxidant enzymes in the Prdx system. Thus, Prdx3 was selected for further analysis.

We performed quantitative real-time PCR and western blotting to confirm this finding. Consistent with our MS results, AZA induced higher Prdx2 and Prdx3 RNA and protein expressions (Figures 4(b) and 4(d)). Finally, we used STRING to analyze the proteins that interacted with Prdx3 and found that Prdx3 interacted with many antioxidant enzymes, including catalase (CAT) and SOD2 (Figure 4(c)), which were also upregulated after AZA treatment as shown in Figure 4(a). This result was also validated by further analysis by using RT-PCR and WB in Fig. S3. This unexpected finding indicated that the upregulation of SOD2 and CAT caused by AZA may have a synergetic effect with Prdx3 on decreasing intracellular ROS levels. Many nutraceuticals and antioxidants, such as vitamin C, selenium, and lycopene, have been developed for cancer prevention and treatment as they can scavenge ROS [14]; likewise, AZA can exert antileukemic effects by decreasing ROS levels via upregulation of Prdx2 and Prdx3.

### 3.5. AZA Repressed the Leukemic Growth In Vivo

**Figure 5:**
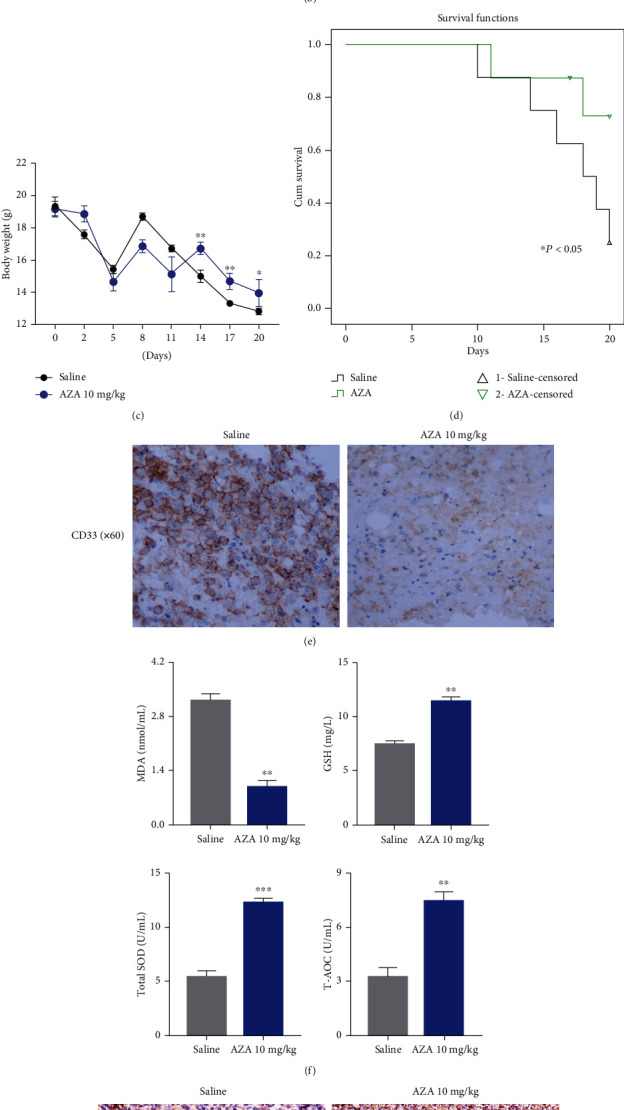
AZA suppressed AML tumorigenicity *in vivo*. (a) Flow chart of the PDX animal model construction and treatment. (b) AML patient cells could be observed on the peripheral blood (PB) and bone marrow (BM) smears under a microscope. Magnification: ×100. (c) Body weight change. (d) Survival. There were 8 mice in each group. (e) The expression of CD33 in mice BM after AZA treatment and its detection by immunohistochemistry. Magnification: ×40. (f) The levels of ROS-related indices MDA, SOD, GSH, and T-AOC in blood plasma after AZA treatment. (g) The expression of Prdx2 and Prdx3 in mice spleen after AZA treatment and their detection by immunohistochemistry. Magnification: ×40. ^∗^*P* < 0.05, ^∗∗^*P* < 0.01, and ^∗∗∗^*P* < 0.001.

To test whether AZA could repress leukemia in *vivo*, we constructed a PDX AML model as per a previous report [40]. Briefly, B-NSG mice were intravenously injected with 1.0 × 10^7^ CD33^+^ AML patient primary cells after 1.5 Gy irradiation. When PB and BM smears indicated illness, mice were randomly divided into two groups (*n* = 8). AZA was administered every three days for 2 weeks; saline was administered as a negative control. The PDX AML models progressed rapidly, and most mice died within 18-27 days; thus, all mice were sacrificed on day 21, and their tissues were harvested for further study (Figure 5(a)). AML patient cells could be observed on the PB and BM smears one week after the injection, and the leukemic blasts could be observed on the BM and spleen by hematoxylin and eosin stain, which indicated the development of leukemia disease (Figures 5(b) and S4). Mice in the AZA group lost weight more slowly and survive longer than did mice in the saline group (Figures 5(c) and 5(d)). Additionally, the percentage of CD33^+^ AML cells in the BM was significantly decreased in the AZA group compared with the saline group, suggesting disease remission (Figure 5(e)). Importantly, compared with the saline group, the MDA levels were decreased, while the T-AOC, GSH levels, and SOD activity were increased in the AZA group in the mouse blood plasma (Figure 5(f)). Moreover, higher Prdx2 and Prdx3 expressions occurred in the AZA group than in the saline group on BM biopsies detected via immunohistochemical staining in the PDX AML model (Figure 5(g)). These results were consistent with the *in vitro* experimental results.

These results suggest that AZA decreases intracellular ROS levels and increases the antioxidant capacity by upregulating Prdx2 and Prdx3, thus maintaining the redox balance and further suppressing AML *in vitro* and *in vivo.*

## 4. Discussion

AML has a high incidence of relapse and poor prognosis, one of the main reasons is therapeutic resistance. Although emerging targeted drugs and immunotherapies may benefit some patients, they are limited in their applications. Tyrosine kinase inhibitors (TKI) can be used only in patients with FLT-3 mutations and patients who develop resistance [41]. Immune checkpoint inhibitors (ICIs) are effective in malignancies with high mutational burden, but ICIs have little effect on AML because AML patients have the lowest mutational burden [42]. Chimeric antigen receptor T-cell (CAR-T) therapy also has a modest effect on AML because of the lack of leukemia-specific cell surface antigens [43, 44]. Therefore, novel agents and therapeutic approaches should be exploited.

ROS are generated from multiple sources. The main source of intracellular ROS is the mitochondria. Healthy cells control the intracellular ROS balance via the scavenging system [45]. One major scavenging system by which mitochondria neutralize excess ROS is through a dedicated Prdx system comprising Prdx3. Prdx3 is exclusively located in the mitochondria. Prdx3 is the most abundant and efficient H_2_O_2_-eliminating enzyme involved in detoxifying 90% of H_2_O_2_ [8]. Deleting of Prdx3 results in H_2_O_2_ accumulation and elevated ROS levels in the mitochondria [46]. Prdx2 is a cytoplasmic Cys-dependent peroxidase with the highest affinity of H_2_O_2_ [45]. Early studies showed that Prdx2 inhibited the myeloid cell proliferation by reducing ROS levels and acted as an epigenetically silenced tumor suppressor in AML [47]. In addition, activation of Prdx2 by depletion of cyclin-dependent kinase 2 (CDK2) can drive the therapeutic differentiation of AML [48]. Moreover, Prdx3 and Prdx2 have been associated with cancer aggressiveness and patient survival, and higher Prdx2 and Prdx3 expressions were associated with less aggressiveness and longer survival [47, 49]. In our study, we identified that AZA increased Prdx2 and Prdx3 expressions, thus regulating the redox state by decreasing ROS levels to suppress AML growth.

Identifying agents that can decrease intracellular ROS levels is a potential method of treating cancer. Lycopene can suppress the progression of prostate carcinoma by decreasing oxidative DNA damage and scavenging ROS [50, 51]. Some clinical trials showed selenium and vitamin C supplementation could decrease the incidence and mortality of gastric and lung cancer [52, 53]. One study demonstrated selenium exerted antileukemia effect by increasing antioxidant capacity [54]. A previous study showed that AZA exhibited antitumor effects against melanoma by inhibiting ROS generation and reducing oxidative tissue injury [28]. In the present study, AZA inhibited AML proliferation by decreasing intracellular ROS levels and increasing the antioxidant capacity. Further analysis of the mechanisms by quantitative proteomics, qPCR, western blot, and immunohistochemistry identified that antioxidative Prdx2 and Prdx3 were upregulated after AZA treatment. Accordingly, we concluded that AZA exerts antileukemic effects by regulating the Prdxs/ROS signaling pathway.

AZA exerts antitumor effects at micromole concentration, it was usually topical application in the form of water-miscible or gel for the treatment of skin disorder with only mild additional side effects; however, when it was administrated orally or intraperitoneal injection in the form of solution, it could cause noticeable weight loss and acid-base imbalance [55, 56]. Therefore, further research work is still needed to decrease the required concentration so as to minimize side effects.

Our MS detection results showed that AZA upregulated other antioxidant enzymes such as CAT and SOD2. These antioxidant enzymes interact with Prdx3 and Prdx2, but how they act in synergy with Prdxs to maintain intracellular redox equilibrium is being studied. Several immune-related signaling pathways were active after AZA treatment as shown on the heat map; the most obvious was Notch. Notch can maintain low ROS levels to promote cell development and survival [57]. In addition, Notch is an important regulator of immune cell development and function [58]. Our previous study had identified that AZA could activate the Notch signaling pathway and enhance the cytotoxicity of immune effector cells against AML [59]. However, whether AZA can alleviate immunosuppression in the tumor microenvironment via the regulation of Prdxs and Notch requires further study.

In summary, we provided a potential novel agent and therapeutic approach to treating AML by regulating the Prdxs/ROS signaling pathway, which may provide insight into ROS-eliminating strategies.

## 5. Conclusion

AZA decreased intracellular ROS levels and increased antioxidant capacity by upregulating Prdx2 and Prdx3, which maintained the intracellular redox balance and further suppressed AML *in vitro* and *in vivo*. AZA is a potential agent for treating AML; ROS-eliminating strategies may be promising strategies for treating AML.

## Data Availability

The raw data supporting the conclusions of this manuscript will be made available by the authors.

## Supplementary Materials

Supplementary Materials Figure S1: AML cell lines and different types of AML patient cells were treated with different concentrations of AZA for 24 h. Cell viability was measured by the CCK-8 method. Figure S2: cell apoptosis rate was analyzed by flow cytometry after healthy PBMCs were treated with 5.0 mM AZA for 24 h. Figure S3: the expression levels of SOD2 and CAT in Molm-13 and THP-1 cells after AZA treatment and their detection by RT-PCR (A) and western blot (B). SOD2 (Cat# 24127-1-AP) and CAT (Cat# 21260-1-AP) used for WB were from ProteinTech (USA). The primers for RT-PCR are as follows: SOD2 (MnSOD): forward 5′-GCC TCC CTG ACC TGC CTT AC-3′, reverse 5′-GCA TGA TCT GCG CGT TAA TG-3′; CAT (catalase): forward 5′-CCC AGA AGC CTA AGA ATG CAA-3′, reverse 5′-GCT TTT CCC TTG GCA GCT ATG-3. Figure S4: AML patient cells could be observed on the bone marrow (BM) and spleen smears by hematoxylin and eosin stain under a microscope. Magnification: ×60. Table S1: the detailed genetic information of AML patient cells.

## References

[1] Beyar-Katz O., Gill S. (2018). Novel approaches to acute myeloid leukemia immunotherapy. *Clinical Cancer Research*.

[2] Nathan C., Cunningham-Bussel A. (2013). Beyond oxidative stress: an immunologist’s guide to reactive oxygen species. *Nature Reviews Immunology*.

[3] Sies H., Jones D. P. (2020). Reactive oxygen species (ROS) as pleiotropic physiological signalling agents. *Nature reviews Molecular Cell Biology*.

[4] Liou G. Y., Storz P. (2010). Reactive oxygen species in cancer. *Free Radical Research*.

[5] Popp H. D., Bohlander S. K. (2010). Genetic instability in inherited and sporadic leukemias. *Genes, Chromosomes & Cancer*.

[6] Trachootham D., Alexandre J., Huang P. (2009). Targeting cancer cells by ROS-mediated mechanisms: a radical therapeutic approach?. *Nature Reviews Drug Discovery*.

[7] Zhou F. L., Zhang W. G., Wei Y. C. (2010). Involvement of oxidative stress in the relapse of acute myeloid leukemia. *The Journal of Biological Chemistry*.

[8] Hole P. S., Zabkiewicz J., Munje C. (2013). Overproduction of NOX-derived ROS in AML promotes proliferation and is associated with defective oxidative stress signaling. *Blood*.

[9] Hole P. S., Pearn L., Tonks A. J. (2010). Ras-induced reactive oxygen species promote growth factor-independent proliferation in human CD34+ hematopoietic progenitor cells. *Blood*.

[10] Zhou F., Shen Q., Claret F. X. (2013). Novel roles of reactive oxygen species in the pathogenesis of acute myeloid leukemia. *Journal of Leukocyte Biology*.

[11] Zhou F., Pan Y., Wei Y. (2017). Jab1/Csn5-thioredoxin signaling in relapsed acute monocytic leukemia under oxidative stress. *Clinical cancer research : an official journal of the American Association for Cancer Research*.

[12] Ito K., Hirao A., Arai F. (2006). Reactive oxygen species act through p38 MAPK to limit the lifespan of hematopoietic stem cells. *Nature Medicine*.

[13] Li X., Fang P., Mai J., Choi E. T., Wang H., Yang X. F. (2013). Targeting mitochondrial reactive oxygen species as novel therapy for inflammatory diseases and cancers. *Journal of Hematology & Oncology*.

[14] Gupta S. C., Hevia D., Patchva S., Park B., Koh W., Aggarwal B. B. (2012). Upsides and downsides of reactive oxygen species for cancer: the roles of reactive oxygen species in tumorigenesis, prevention, and therapy. *Antioxidants & Redox Signaling*.

[15] Vivas-Mejia P. E., Ozpolat B., Chen X., Lopez-Berestein G. (2009). Downregulation of the c-MYC target gene, peroxiredoxin III, contributes to arsenic trioxide-induced apoptosis in acute promyelocytic leukemia. *International Journal of Cancer*.

[16] Bera R., Chiu M. C., Huang Y. J., Liang D. C., Lee Y. S., Shih L. Y. (2018). Genetic and epigenetic perturbations by DNMT3A-R882 mutants impaired apoptosis through augmentation of PRDX2 in myeloid leukemia cells. *Neoplasia*.

[17] Liu C. X., Yin Q. Q., Zhou H. C. (2012). Adenanthin targets peroxiredoxin I and II to induce differentiation of leukemic cells. *Nature Chemical Biology*.

[18] Sotgia F., Martinez-Outschoorn U. E., Lisanti M. P. (2011). Mitochondrial oxidative stress drives tumor progression and metastasis: should we use antioxidants as a key component of cancer treatment and prevention?. *BMC Medicine*.

[19] Bossis G., Sarry J. E., Kifagi C. (2014). The ROS/SUMO axis contributes to the response of acute myeloid leukemia cells to chemotherapeutic drugs. *Cell Reports*.

[20] Gaballa S., Saliba R., Oran B. (2017). Relapse risk and survival in patients with FLT3 mutated acute myeloid leukemia undergoing stem cell transplantation. *American Journal of Hematology*.

[21] Sallmyr A., Fan J., Datta K. (2008). Internal tandem duplication of FLT3 (FLT3/ITD) induces increased ROS production, DNA damage, and misrepair: implications for poor prognosis in AML. *Blood*.

[22] Sarhan D., Wang J., Arvindam U. S. (2020). Mesenchymal stromal cells shape the MDS microenvironment by inducing suppressive monocytes that dampen NK cell function. *JCI Insight*.

[23] Weinberg S. E., Sena L. A., Chandel N. S. (2015). Mitochondria in the regulation of innate and adaptive immunity. *Immunity*.

[24] Breathnach A. S., Robins E. J., Pätzold H. C. (1989). Effect of dicarboxylic acids (C6 and C9) on human choroidal melanoma in cell culture. *Investigative Ophthalmology & Visual Science*.

[25] Nazzaro-Porro M., Passi S. (1978). Identification of tyrosinase inhibitors in cultures of Pityrosporum. *The Journal of Investigative Dermatology*.

[26] Schallreuter K. U., Wood J. M. (1987). Azelaic acid as a competitive inhibitor of thioredoxin reductase in human melanoma cells. *Cancer Letters*.

[27] Passi S., Picardo M., Zompetta C., De Luca C., Breathnach A. S., Nazzaro-Porro M. (2009). The oxyradical-scavenging activity of azelaic acid in biological systems. *Free Radical Research Communications*.

[28] Akamatsu H., Komura J., Asada Y., Miyachi Y., Niwa Y. (1991). Inhibitory effect of azelaic acid on neutrophil functions: a possible cause for its efficacy in treating pathogenetically unrelated diseases. *Archives of Dermatological Research*.

[29] Passi S., Picardo M., Nazzaro-Porro M., Breathnach A., Confaloni A. M., Serlupi-Crescenzi G. (1984). Antimitochondrial effect of saturated medium chain length (C_8_-C_13_) dicarboxylic acids. *Biochemical Pharmacology*.

[30] Nazzaro-Porro M. (1987). Azelaic acid. *Journal of the American Academy of Dermatology*.

[31] Leibl H., Stingl G., Pehamberger H., Korschan H., Konrad K., Wolff K. (1985). Inhibition of DNA synthesis of melanoma cells by azelaic acid. *The Journal of Investigative Dermatology*.

[32] Picardo M., Passi S., Sirianni M. C. (1985). Activity of azelaic acid on cultures of lymphoma- and leukemia-derived cell lines, normal resting and stimulated lymphocytes and 3T3 fibroblasts. *Biochemical Pharmacology*.

[33] Ut Y., Furuke K., Masutani H., Nakamura H., Yodoi J. (1995). Cell cycle inhibition of HTLV-I transformed T cell lines by retinoic acid: the possible therapeutic use of thioredoxin reductase inhibitors. *Oncology Research*.

[34] Pan Y., Liu D., Wei Y. (2017). Azelaic acid exerts antileukemic activity in acute myeloid leukemia. *Frontiers in Pharmacology*.

[35] Liang L., Zuo Y. F., Wu W., Zhu X. Q., Yang Y. (2016). Optofluidic restricted imaging, spectroscopy and counting of nanoparticles by evanescent wave using immiscible liquids. *Lab on a Chip*.

[36] Liang L., Jin Y. X., Zhu X. Q., Zhou F. L., Yang Y. (2018). Real-time detection and monitoring of the drug resistance of single myeloid leukemia cells by diffused total internal reflection. *Lab on a Chip*.

[37] Boersema P. J., Raijmakers R., Lemeer S., Mohammed S., Heck A. J. (2009). Multiplex peptide stable isotope dimethyl labeling for quantitative proteomics. *Nature Protocols*.

[38] Jin Y., Yang Q., Liang L. (2018). Compound kushen injection suppresses human acute myeloid leukaemia by regulating the Prdxs/ROS/Trx1 signalling pathway. *Journal of Experimental & Clinical Cancer Research*.

[39] Hall A., Karplus P. A., Poole L. B. (2009). Typical 2-Cys peroxiredoxins--structures, mechanisms and functions. *The FEBS Journal*.

[40] Zhang D., Liu Y., Luo Z. (2020). The novel thioredoxin reductase inhibitor A-Z2 triggers intrinsic apoptosis and shows efficacy in the treatment of acute myeloid leukemia. *Free Radical Biology & Medicine*.

[41] Wu M., Li C., Zhu X. (2018). FLT3 inhibitors in acute myeloid leukemia. *Journal of Hematology & Oncology*.

[42] Alexandrov L. B., Initiative A. P. C. G., Nik-Zainal S. (2013). Signatures of mutational processes in human cancer. *Nature*.

[43] Gill S., Tasian S. K., Ruella M. (2014). Preclinical targeting of human acute myeloid leukemia and myeloablation using chimeric antigen receptor-modified T cells. *Blood*.

[44] Jetani H., Garcia-Cadenas I., Nerreter T. (2018). CAR T-cells targeting FLT3 have potent activity against FLT3^−^ITD^+^ AML and act synergistically with the FLT3-inhibitor crenolanib. *Leukemia*.

[45] Winterbourn C. C. (2008). Reconciling the chemistry and biology of reactive oxygen species. *Nature Chemical Biology*.

[46] Chang T. S., Cho C. S., Park S., Yu S., Kang S. W., Rhee S. G. (2004). Peroxiredoxin III, a mitochondrion-specific peroxidase, regulates apoptotic signaling by mitochondria. *The Journal of Biological Chemistry*.

[47] Agrawal-Singh S., Isken F., Agelopoulos K. (2012). Genome-wide analysis of histone H3 acetylation patterns in AML identifies PRDX2 as an epigenetically silenced tumor suppressor gene. *Blood*.

[48] Ying M., Shao X., Jing H. (2018). Ubiquitin-dependent degradation of CDK2 drives the therapeutic differentiation of AML by targeting PRDX2. *Blood*.

[49] Luthra S., Chandran U., Diergaarde B., Becich M., Lee A. V., Neumann C. A. (2018). Expression of reactive species related genes is associated with patient survival in luminal B breast cancer. *Free Radical Biology & Medicine*.

[50] Chen L., Stacewicz-Sapuntzakis M., Duncan C. (2001). Oxidative DNA damage in prostate cancer patients consuming tomato sauce-based entrees as a whole-food intervention. *Journal of the National Cancer Institute*.

[51] Kim H. S., Bowen P., Chen L. (2003). Effects of tomato sauce consumption on apoptotic cell death in prostate benign hyperplasia and carcinoma. *Nutrition and Cancer*.

[52] Duffield-Lillico A. J., Reid M. E., Turnbull B. W. (2002). Baseline characteristics and the effect of selenium supplementation on cancer incidence in a randomized clinical trial: a summary report of the Nutritional Prevention of Cancer Trial. *Cancer Epidemiology and Prevention Biomarkers*.

[53] Li W.-Q., Zhang J.-Y., Ma J.-L. (2019). Effects of Helicobacter pylori treatment and vitamin and garlic supplementation on gastric cancer incidence and mortality: follow-up of a randomized intervention trial. *BMJ*.

[54] Jin Y., Cai L., Yang Q. (2020). Anti-leukemia activities of selenium nanoparticles embedded in nanotube consisted of triple-helix *β*-d-glucan. *Carbohydrate Polymers*.

[55] Wu C., Hwang S. H., Jia Y. (2017). Olfactory receptor 544 reduces adiposity by steering fuel preference toward fats. *The Journal of Clinical Investigation*.

[56] Bojar R. A., Cunliffe W. J., Holland K. T. (1994). Disruption of the transmembrane pH gradient--a possible mechanism for the antibacterial action of azelaic acid in Propionibacterium acnes and Staphylococcus epidermidis. *The Journal of Antimicrobial Chemotherapy*.

[57] Small C., Ramroop J., Otazo M., Huang L. H., Saleque S., Govind S. (2014). An unexpected link between notch signaling and ROS in restricting the differentiation of hematopoietic progenitors in Drosophila. *Genetics*.

[58] Radtke F., MacDonald H. R., Tacchini-Cottier F. (2013). Regulation of innate and adaptive immunity by Notch. *Nature Reviews Immunology*.

[59] Dongdong Z., Jin Y., Yang T. (2019). Antiproliferative and immunoregulatory effects of azelaic acid against acute myeloid leukemia via the activation of Notch signaling pathway. *Frontiers in Pharmacology*.

