# Supplementary material for: Azelaic Acid Exerts Antileukemia Effects against Acute Myeloid Leukemia by Regulating the Prdxs/ROS Signaling Pathway
Source: Oxid Med Cell Longev. 2020 Dec 23;2020:1295984. doi: 10.1155/2020/1295984 (PMC7775164; doi:10.1155/2020/1295984)
Supplement: Supplementary Materials — Figure S1: AML cell lines and different types of AML patient cells were treated with different concentrations of AZA for 24 h. Cell viability was measured by the CCK-8 method. Figure S2: cell apoptosis rate was analyzed by flow cytometry after healthy PBMCs were treated with 5.0 mM AZA for 24 h. Figure S3: the expression levels of SOD2 and CAT in Molm-13 and THP-1 cells after AZA treatment and their detection by RT-PCR (A) and western blot (B). SOD2 (Cat# 24127-1-AP) and CAT (Cat# 21260-1-AP) used for WB were from ProteinTech (USA). The primers for RT-PCR are as follows: SOD2 (MnSOD): forward 5′-GCC TCC CTG ACC TGC CTT AC-3′, reverse 5′-GCA TGA TCT GCG CGT TAA TG-3′; CAT (catalase): forward 5′-CCC AGA AGC CTA AGA ATG CAA-3′, reverse 5′-GCT TTT CCC TTG GCA GCT ATG-3. Figure S4: AML patient cells could be observed on the bone marrow (BM) and spleen smears by hematoxylin and eosin stain under a microscope. Magnification: ×60. Table S1: the detailed genetic information of AML patient cells. [file 1295984.f1.pdf]

Azelaic acid exerts antileukemia effects against acute myeloid leukemia by regulating the Prdxs/ROS signaling pathway

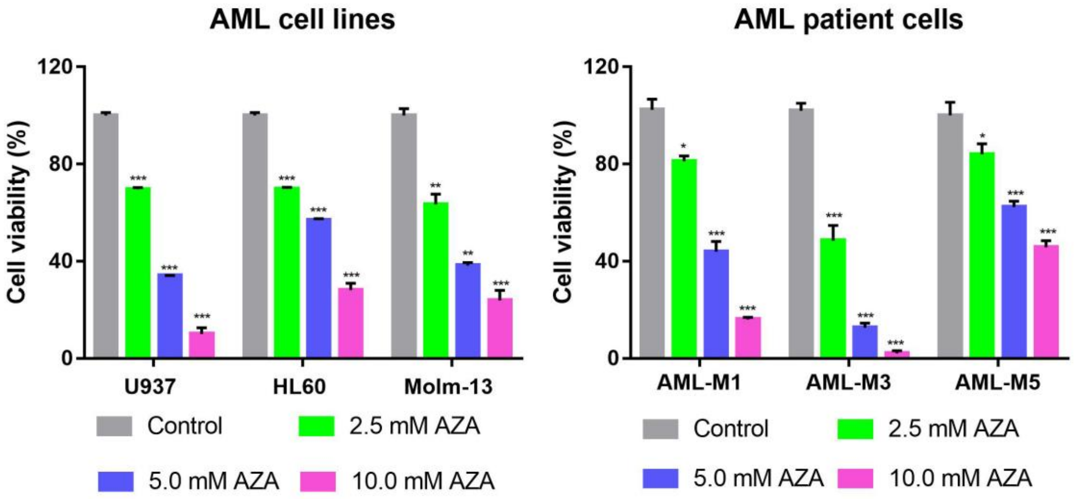

Figure S1. AML cell lines and different types of AML patient cells were treated with different concentration of AZA for 24 h. Cell viability was measured by the CCK-8 method.

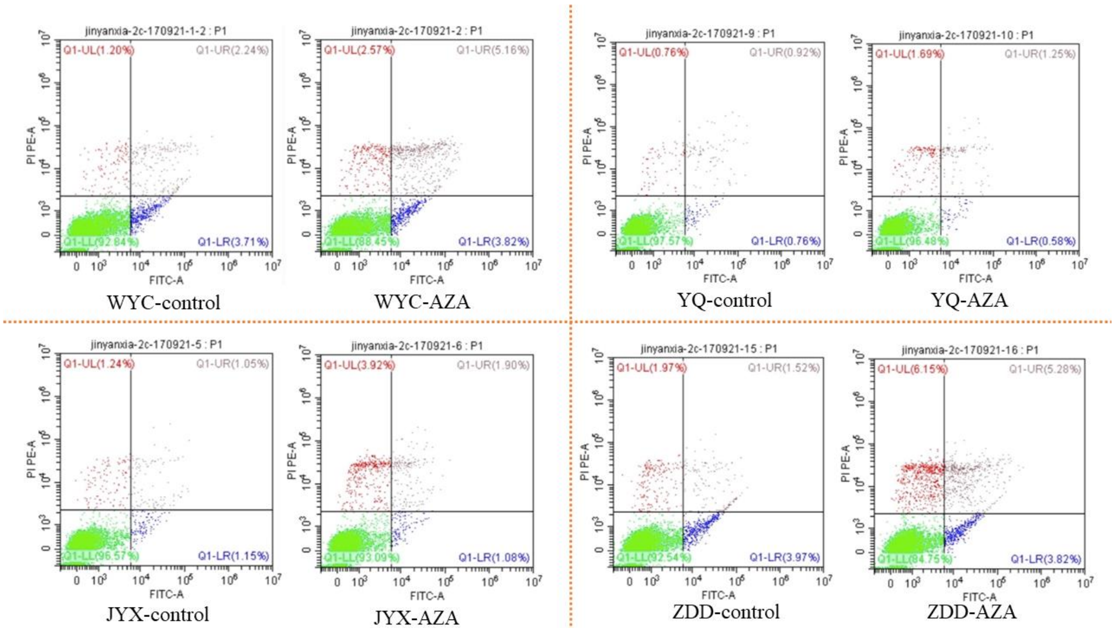

Figure S2. Cell apoptosis rate was analyzed by flow cytometry after healthy PBMCs were treated with 5.0 mM AZA for 24 h.

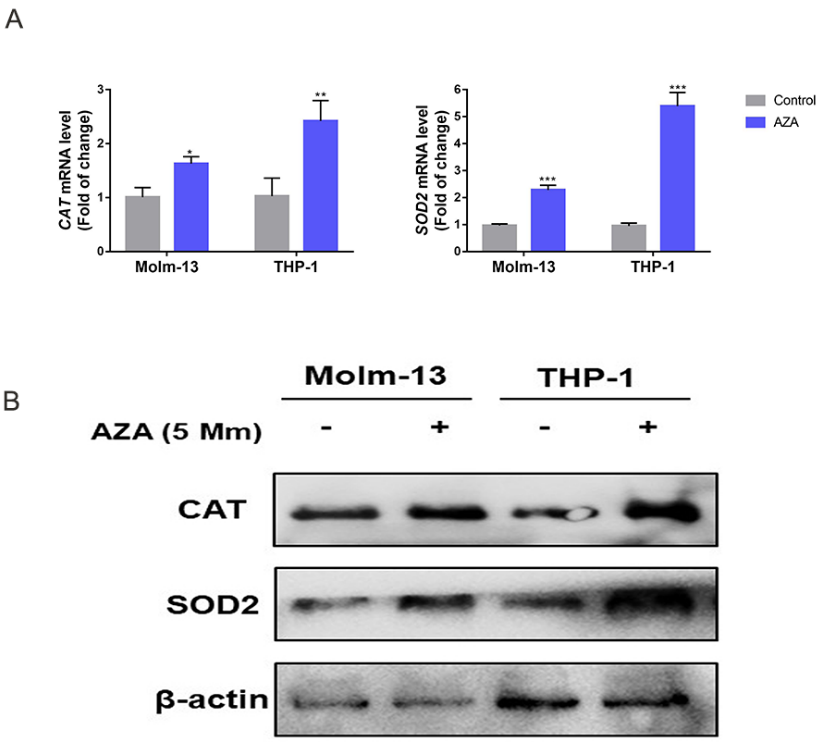

Figure S3. The expression levels of SOD2 and CAT in Molm-13 and THP-1 cells after AZA treatment and their detection by RT-PCR (A) and western blot (B) **.** SOD2 (Cat# 24127-1-AP) and CAT (Cat# 21260-1-AP) used for WB were from ProteinTech (USA). The Primers for RT-PCR are as followed: SOD2 (*MnSOD)*: forward 5′-GCC TCC CTG ACC TGC CTT AC-3′, reverse 5′-GCA TGA TCT GCG CGT TAA TG-3′;

CAT (*Catalase*) : forward 5′-CCC AGA AGC CTA AGA ATG CAA-3′,

reverse 5′-GCT TTT CCC TTG GCA GCT ATG-3

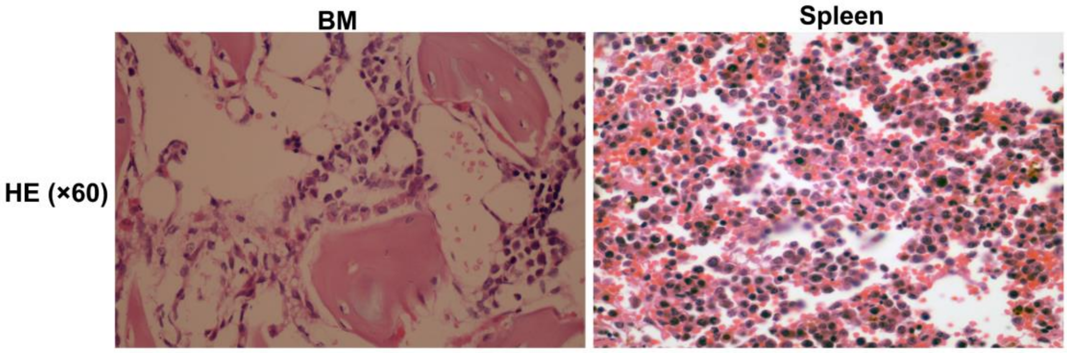

Figure S4. AML patient cells could be observed on the bone marrow (BM) and spleen smears by hematoxylin and eosin stain under a microscope. Magnification: ×60.

Type	Genetic mutation
AML-M1	CEBPA, FLT3-ITD mutation
AML-M3	None
AML-M5 (it was also used for construction of PDX model)	FLT3-ITD,NPM1L mutation<br>Immunophenotyping:<br>express CD64, CD117,CD33,CD123;<br>some express: MPO, CD56,CD13

Table S1. The detailed genetic information of AML patient cells.

We uploaded the raw LC-MS data on PeptideAtlas according to the author's guideline. The direct URL is http://www.peptideatlas.org/PASS/PASS01499 and the password is JG6834ntz.
